# Predictive Study of the Active Ingredients and Potential Targets of *Codonopsis pilosula* for the Treatment of Osteosarcoma via Network Pharmacology

**DOI:** 10.1155/2021/1480925

**Published:** 2021-06-04

**Authors:** Yu-Bao Gong, Shao-Jie Fu, Zheng-Ren Wei, Jian-Guo Liu

**Affiliations:** ^1^Department of Orthopedics, The First Hospital, Jilin University, Changchun 130021, China; ^2^Department of Nephrology, The First Hospital, Jilin University, Changchun 130021, China; ^3^Department of Pharmacology, The Basic Medical School, Jilin University, Changchun, Jilin 130021, China

## Abstract

Osteosarcoma (OS) is the most common type of primary bone tumor in children and adults. Dangshen (*Codonopsis pilosula*) is a traditional Chinese medicine commonly used in the treatment of OS worldwide. However, the molecular mechanisms of Dangshen in OS remain unclear. Hence, in this study, we aimed to systematically explore the underlying mechanisms of Dangshen in the treatment of OS. Our study adopted a network pharmacology approach, focusing on the identification of active ingredients, drug target prediction, gene collection, gene ontology (GO) enrichment, Kyoto Encyclopedia of Genes and Genomes (KEGG) enrichment, and other network tools. The network analysis identified 15 active compounds in Dangshen that were linked to 48 possible therapeutic targets related to OS. The results of the gene enrichment analysis show that Dangshen produces a therapeutic effect in OS likely by regulating multiple pathways associated with DNA damage, cell proliferation, apoptosis, invasion, and migration. Based on the network pharmacology approach, we successfully predicted the active compounds and their respective targets. In addition, we illustrated the molecular mechanisms that mediate the therapeutic effect of Dangshen in OS. These findings may aid in the development of novel targeted therapies for OS in the future.

## 1. Introduction

Osteosarcoma (OS) is the most common type of cancer of the bones. It is a malignant tumor that primarily affects the long bones (e.g., legs), but it can also start in other bones. OS is rarely diagnosed in patients under five years of age, and the bimodal age-incidence curve peaks during the second decade of life (10–20 years old) and late adulthood (>40 years old) [1, 2]. As of 2019, approximately 560 children and adolescents are affected each year in the United States [1, 3], with a global incidence of 3.4 cases per one million people. Most OS patients present with metastatic disease, which contributes to its high morbidity and mortality rates worldwide. As of 2020, the standard treatment for OS is systemic chemotherapy [4], as the tumor is often resistant to radiation therapy. Surgical resection may be an option for patients diagnosed with locally noninvasive disease [5]. Most patients undergo multifaceted treatments that include preoperative chemotherapy, postoperative chemotherapy, surgical resection, and radiation therapy in rare cases [6]. Detectable metastases are present in only 20% of patients, and most of the remaining 80% of patients have undetectable micrometastases [7]. This makes it challenging to monitor disease progression and treatment response, which is why many physicians rely on long-term systemic chemotherapy [8]. However, the systemic chemotherapeutics needed to control the disease have serious adverse effects that further hinder the effective treatment of patients in the clinic [9].

In recent years, some hospitals have assessed the efficacy of traditional Chinese medicines as long-term treatments for OS. In some cases, orally administered Shenqi could decrease the growth, metastasis, and the number of chemotherapy-related side effects significantly in patients, especially those who received systemic chemotherapy [10, 11]. The major component of the Shenqi oral preparation is Dangshen, also known as (*Codonopsis pilosula*). Dangshen belongs to the family of Campanulaceae, a precious plant that grows at altitudes of 2000 meters in southern China [12]. The dry roots of this plant have been used for thousands of years in traditional Chinese medicine to treat qi and blood deficiencies, the loss of appetite, respiratory symptoms (e.g., cough, asthma, and shortness of breath), and cardiovascular problems (e.g., palpitations) [13]. Dangshen displays a variety of pharmacological effects on the circulatory system, immune system, digestive system, endocrine system, and reproductive system [14]. Dangshen has been shown to inhibit cancer growth in S180 tumor-bearing mice, while enhancing the immune response, increasing spleen weight, promoting lymphocyte proliferation, and increasing natural killer (NK) cell activity [15, 16].

DNA damaging therapies are widely used as trigger molecules to study the signaling pathways of OS [17]. There has been a remarkable undertaking of investigations into the different signaling pathways involved in the pathogenesis of OS. Many signaling pathways, such as Wnt, PI3K/AKT, and JAK/STAT, reflect their specific roles in OS [18]. However, conventional research methods have been unable to fully elucidate the mechanisms of action. Nevertheless, the integration of bioinformatics and network pharmacology provides a practical approach to explore and verify the mechanisms of action [19]. Network pharmacology can systematically reveal the active components in drug molecules. In addition, network pharmacology can be used to predict the relationship between drug components and gene targets [20]. Therefore, in this study, we aimed to use network pharmacology to uncover the mechanisms by which Dangshen produces therapeutic effects in patients with OS, along with the associated signaling pathways.

## 2. Materials and Methods

### 2.1. Chemical Compounds in Dangshen

**Figure 1:**
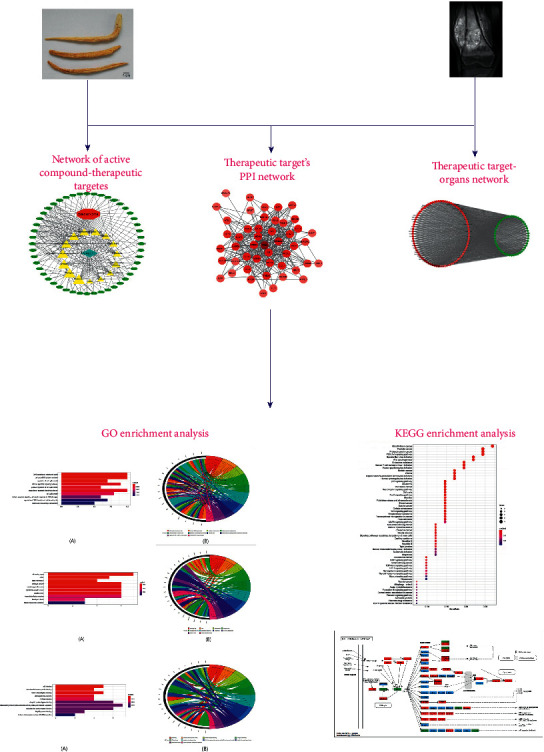
Schematic illustration showing the network pharmacology study of Dangshen (*Codonopsis pilosula*) for the treatment of osteosarcoma (OS).

A flowchart of the study design is shown in Figure 1. The components of Dangshen were searched in the Traditional Chinese Medicine Systems Pharmacology (TCMSP) (http://tcmspw.com/tcmsp.php) database [21] and Traditional Chinese Medicines Integrated Database (TCMID) (http://www.megabionet.org/tcmid/) [22]. TCMSP provides comprehensive information about components in Chinese herbs, while TCMID provides information on all aspects of traditional Chinese medicines, including herbs and herbal ingredients. Oral bioavailability (OB), which is the percentage of an orally administered drug that reaches the systemic circulation, reflects the degree of absorption and utilization of drugs in the body [23]. The drug-likeness (DL) value reflects the structural similarity between the compound and drug molecule, so the DL compounds are more likely to display suitable pharmacodynamic and pharmacokinetic properties [24]. Therefore, we selected the candidate compounds based on OB and DL properties. As suggested by the TCMSP database, OB ≥ 30% and DL ≥ 0.18 were used as the screening criteria, and the compounds whose OB ≥ 30% and DL ≥ 0.18 were selected for subsequent experiments [25]. We searched the oral bioavailability of all the compounds of Dangshen on PubMed. If the OB data of some compounds were previously reported in related experiments, the real-world data of OB were used instead of silicon data, and the highest reported OB value was adopted. Otherwise, silicon data were used for OB. The TCMSP database calculated OB values by using OBioavail1.1. This model shows good potential in facilitating the prediction of oral bioavailability and can be applied in drug design.

### 2.2. Compounds of Dangshen and Their Targets

PubChem (https://pubchem.ncbi.nlm.nih.gov/) is an open chemistry database of the National Institutes of Health (NIH) [26]. This database serves as an important source of chemical information, including chemical structures, biological activities, chemical and physical properties, and safety [27]. We imported the compounds filtered from Dangshen into PubChem and obtained their 3D molecular structure files in SDF format. Structural information is necessary for predicting the targets of compounds, so the compounds without precise structural details were removed from the analysis.

PharmMapper (http://www.lilab-ecust.cn/pharmmapper/check.html) is a freely accessed web server that uses the pharmacophore mapping approach to identify potential small-molecule targets [28, 29]. We imported the 3D structural files in SDF format into PharmMapper and selected the pharmacophore model with a pKd value ≥ 6.0. In our study, the top matched 50 targets were selected as the potential targets of each compound.

### 2.3. Collection of Gene Targets in OS

The human genes associated with OS were gathered from OMIM (Online Mendelian Inheritance in Man, https://omim.org/) and GeneCards (https://www.genecards.org/). OMIM is an authoritative and comprehensive database of human genes and genetic phenotypes [30], while GeneCards is an integrative database that provides information on all predicted and annotated human genes [31]. The search term “osteosarcoma” was used to retrieve the OS targets from both databases.

### 2.4. Therapeutic Targets of Dangshen in OS

We screened the active compounds of Dangshen and obtained their target genes. We also gathered the OS-related genes. The potential therapeutic targets were identified from the shared genes mentioned above.

### 2.5. Protein-Protein Interaction (PPI) Data

The therapeutic targets were imported into the STRING database to obtain their interaction relationship. STRING (https://string-db.org/, version 11.0) is a database that contains known and predicted protein-protein interactions, and it collects the information using bioinformatics strategies [32]. The species were limited to “*Homo sapiens*,” and the PPIs with confidence scores >0.4 were selected for this study.

### 2.6. Target Organs

Data about the organ targets were collected from the BioGPS (http://biogps.org) database. BioGPS is an extensible and customizable genetic annotation portal that enables researchers to acquire distributed genetic annotation resources [33]. To reveal the underlying mechanisms of Dangshen in OS, median gene expression levels were used as the standard to screen for organs with high expression of the therapeutic targets.

### 2.7. Network Construction

The network of active compounds and therapeutic targets was constructed by linking the compounds and therapeutic targets to understand the complex interactions between the compounds of Dangshen and the therapeutic targets of OS. The network of therapeutic targets and organs was established by linking therapeutic targets and their distribution in organs to clarify the relationship between the therapeutic targets and organs with increased expression of the target. The therapeutic targets' PPI network was built by linking the therapeutic targets to their interacting targets. Next, Cytoscape version 3.7.2 (http://www.cytoscape.org/) was used to present the networks mentioned above, which is a software program for network visualization [34]. Lastly, NetworkAnalyzer [35] was used to calculate three topological parameters of each node in the network, including the degree, betweenness centrality, and closeness centrality [36].

### 2.8. GO and KEGG Pathway Enrichment Analysis

To learn more about the role of therapeutic targets involved in the biological process (BP), cell component (CC), and molecular function (MF), we used the Gene Ontology (GO) database (http://geneontology.org/) to clarify the possible biological mechanisms [37]. The Kyoto Encyclopedia of Genes and Genomes (KEGG) (https://www.kegg.jp/) is a database for extracting biological information about functional classification, annotation, and enriched pathways of various genes [38]. In this study, we used an R-package-Bioconductor clusterProfiler to perform the GO and KEGG enrichment analysis. The R-package-Bioconductor clusterProfiler is widely used to automate the biological term classification and enrichment analysis of gene clusters [39].

## 3. Results

### 3.1. Chemical Compounds of Dangshen

Using the keyword search in TCMSP and TCMID, a total of 134 components of Dangshen were identified, including flavonoids, steroids, alkaloids, glycosides, and triterpenes. According to the OB and DL characteristics of the ingredients, 25 screened compounds were chosen for the next experiments. As structural information is essential for predicting the targets of a compound, ten compounds without 3D structural information were discarded. Finally, 15 compounds were determined as possible active compounds whose characteristics are listed in Table 1.

### 3.2. Dangshen Compound Targets

We obtained the top 50 matched targets for each potential active compound from PharmMapper. These targets were regarded as the potential targets of Dangshen (Supplementary Table S1).

### 3.3. Collection of Gene Targets for OS

“Osteosarcoma” was used as the keyword to retrieve the OS targets from OMIM and GeneCards databases. A total of 2,079 genes were retrieved from the two databases (Supplementary Table S2).

### 3.4. Therapeutic Targets of Dangshen for OS

The targeted genes of Dangshen and OS were obtained. Using the shared genes described above, 48 possible therapeutic targets were obtained, and the features are listed in Table 2.

### 3.5. Active Compound-Therapeutic Target Network

**Figure 2:**
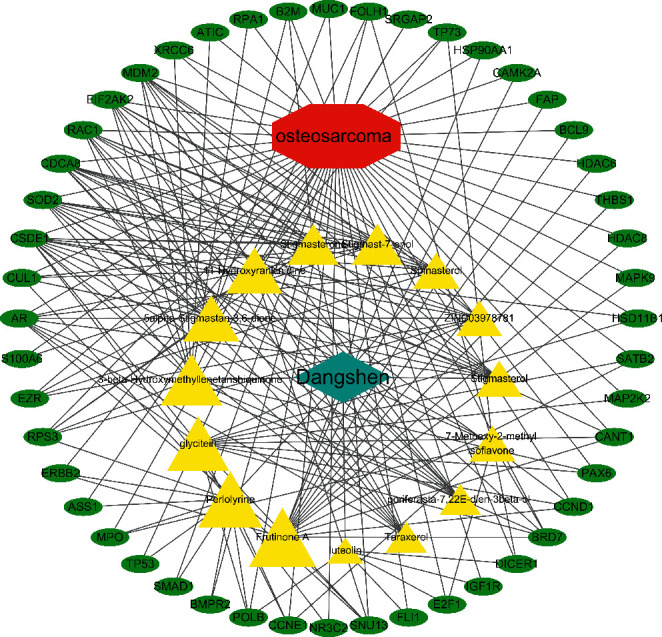
Active compounds-therapeutic targets network. Yellow triangles represent the active compounds from Dangshen, while green ovals represent the therapeutic targets. The size of the triangles is directly proportional to their degree.

The active compound-therapeutic target network is depicted in Figure 2. This network demonstrates the complicated relationship between the compounds and therapeutic targets, including 65 total nodes (15 compound nodes, 48 therapeutic target nodes, one Dangshen node, and one OS node) and 204 edges. In Figure 2, the therapeutic targets are represented by green ovals, Dangshen is represented by a blue quadrangle, OS is represented by a red hexagon, active compounds are represented by yellow triangles, and the sizes of compound nodes were proportional to their degree. The three with the highest degree of the compound nodes were Frutinone A (degree = 16), Perlolyrine (degree = 14), and Glycitein (degree = 13). The three compounds were more likely to show significant therapeutic activity against OS.

### 3.6. Therapeutic Target-PPI Network

**Figure 3:**
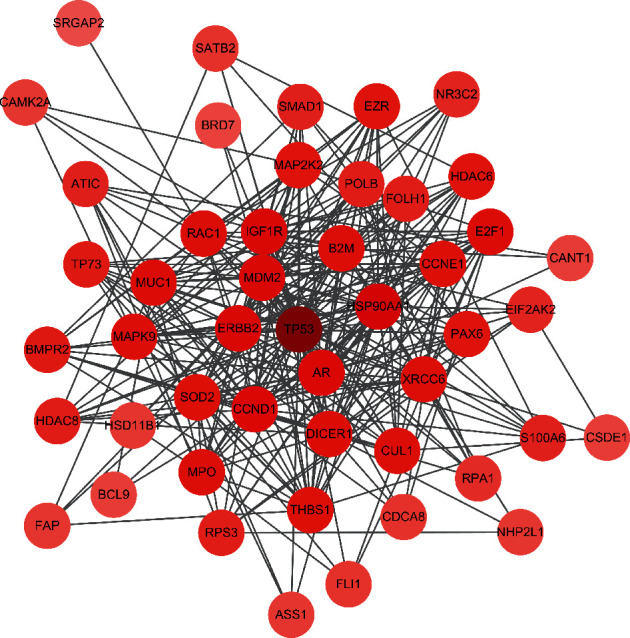
PPI network of therapeutic targets. Hexagons represent the therapeutic targets, and the color shade of hexagons is directly proportional to their degree.

The PPI network of the therapeutic targets is shown in Figure 3, including 48 nodes and 304 edges. NetworkAnalyzer was employed to calculate three topological features of the 48 targets to identify the key nodes in the network (Table 2). The median values of the degree, node betweenness, and closeness were 10, 0.047, and 0.549, respectively. The nodes with “degree >10,” “node betweenness >0.047,” and “node closeness >0.63” were considered to be the key targets. Hence, 20 genes were identified as central targets of Dangshen against OS, including TP53, HSP90AA1, CCND1, AR, ERBB2, MDM2, IGF1R, DICER1, CCNE1, SOD2, among others.

### 3.7. Therapeutic Target-Organ Network

**Figure 4:**
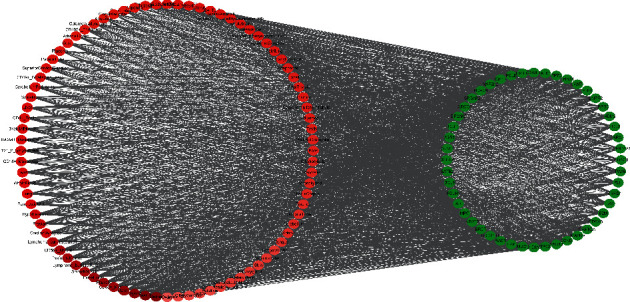
Therapeutic target-organs network. Red ovals represent the tissues with high expression levels of the targets, and the color shade of the red ovals is directly proportional to the degree. Green ovals represent therapeutic targets.

The organs with high expression of each therapeutic target were collected via BioGPS (Supplementary Table S3). The therapeutic target-organ network, shown in Figure 4, is used to delineate the relationship between therapeutic targets and the organs that highly express these targets, including 132 nodes (58 therapeutic target nodes and 84 organ nodes) and 2,031 edges. The color shade of the organ node is proportional to its degree, as shown in Figure 4. These findings demonstrate that many therapeutic targets are highly expressed in tissues, such as the thyroid, retina, pituitary, and pineal gland, and on the surface of antigens, including CD33, CD34, and CD56.

### 3.8. GO and KEGG Pathway Enrichment

**Figure 5:**
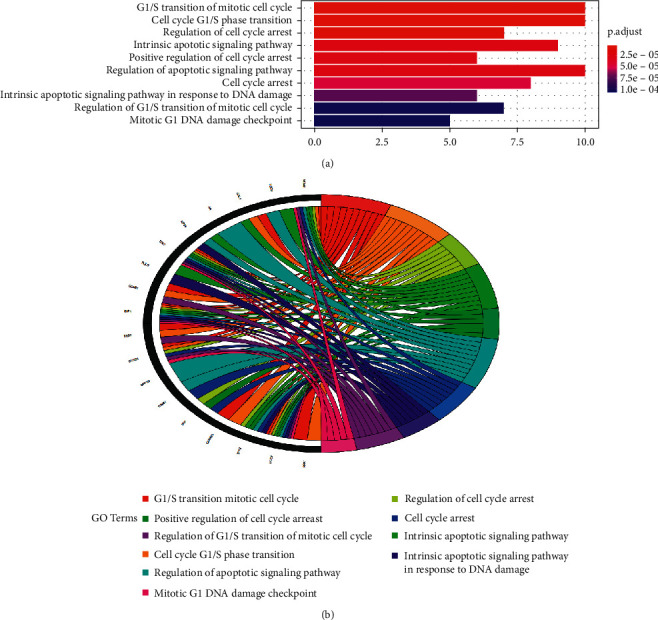
Top ten significant biological process (BP) entries. (a): GO enrichment analysis of therapeutic targets for biological process. (b): Relationship between the therapeutic targets and biological process.

**Figure 6:**
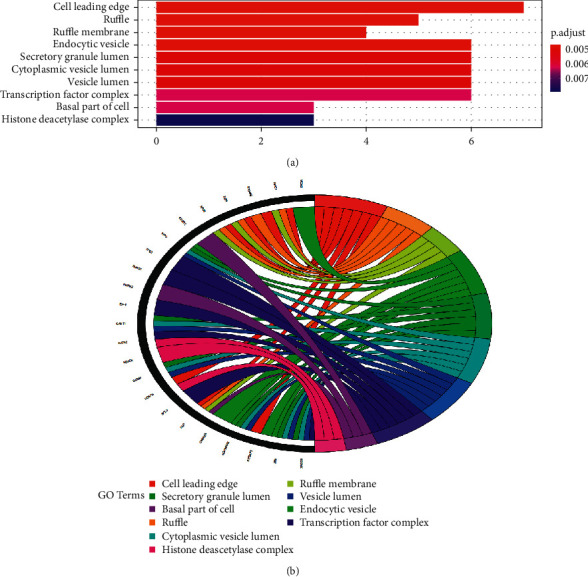
Top ten significant cell component (CC entries). (a): GO enrichment analysis of therapeutic targets for cell components. (b) Relationship between the therapeutic targets and cell components.

**Figure 7:**
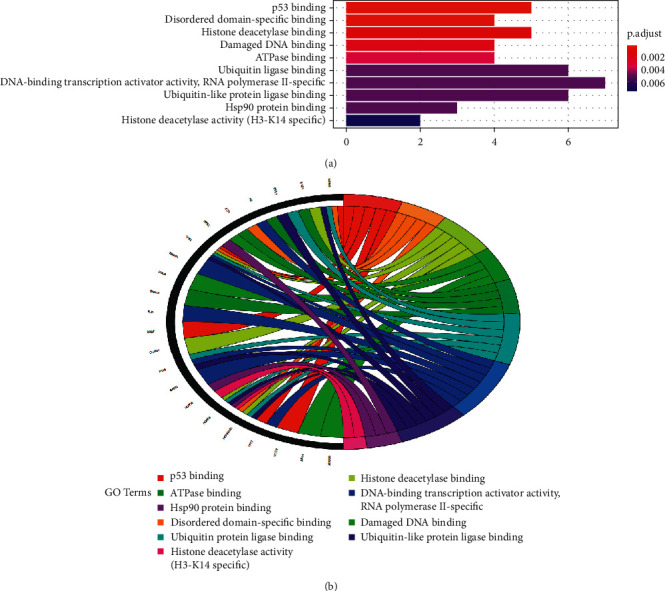
Top ten significant molecular function (MF) entries. (a): GO enrichment analysis of therapeutic targets for molecular function. (b) Relationship between the therapeutic targets and molecular function.

To illuminate the complex mechanisms of Dangshen against OS, we conducted analyses of the GO biological process (BP), cell component (CC), and molecular function (MF) for the 48 therapeutic targets. The top ten biological processes, cell components, and molecular functions are shown in Figures 5(a), 6(a), and 7(a), respectively. The relationship between the genes and biological processes, cell component, and molecular function targets is depicted in Figures 5(b), 6(b), and 7(b), respectively. The details of the GO enrichment analysis of BP, CC, and MF are listed in Supplementary Tables S4–S6, respectively.

**Figure 8:**
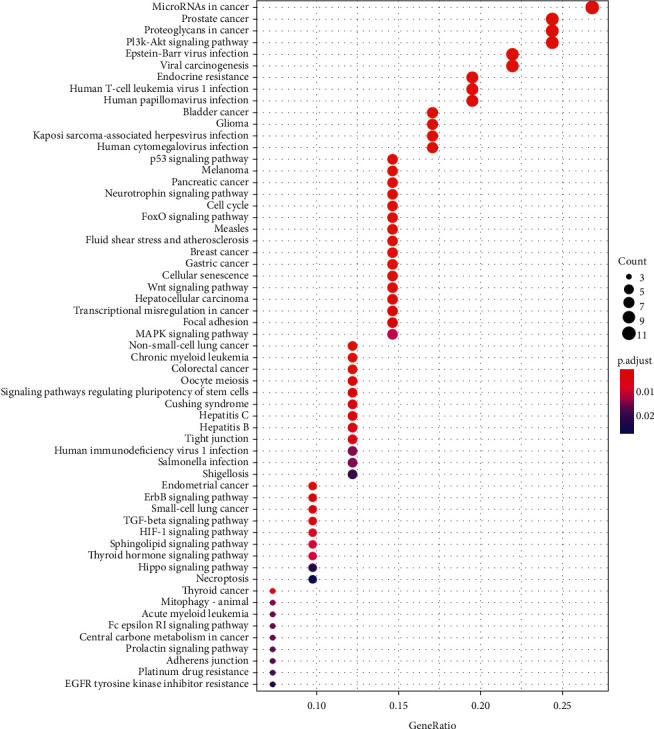
KEGG enrichment analysis for therapeutic targets.

**Figure 9:**
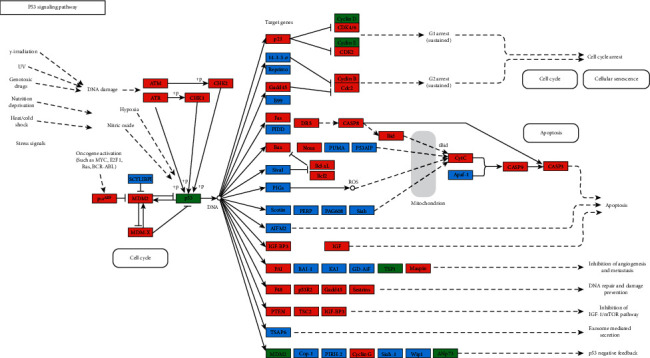
Modulation of the p53 signaling pathway by Dangshen. Targets associated with OS are in red and green, targets of Dangshen are in green, and other proteins in the pathway are in blue.

KEGG pathway enrichment analysis was performed to explore the underlying mechanisms of Dangshen against OS further. As shown in Supplementary Table S7 and Figure 8, there are 69 primary pathways that participate in Dangshen against OS with *p* < 0.05. These 69 pathways involve human diseases, pathophysiological mechanisms, and signaling pathways. The top ten significantly enriched signaling pathways include the p53 signaling pathway, PI3K-Akt signaling pathway, neurotrophin signaling pathway, FoxO signaling pathway, Wnt signaling pathway, ErbB signaling pathway, TGF-*β* signaling pathway, HIF-1 signaling pathway, sphingolipid signaling pathway, and MAPK signaling pathway. Many therapeutic targets are involved in these signaling pathways.  Figure 9 depicts a concept map containing Dangshen and OS targets in the P53 signaling pathway, further demonstrating that Dangshen regulates key targets in this signaling pathway.

## 4. Discussion

Osteosarcoma (OS) is the most common primary bone tumor found in the clinic [40]. It is characterized by high metastatic rates, poor prognoses, and high mortality rates [41]. Dangshen (*Codonopsis pilosula*) is a well-known herbal medicine, and traditional Chinese medicine (TCM) preparation, based on Dangshen, which has shown high efficacy in the treatment of OS [10, 11]. However, its pharmacological mechanisms remain unclear. In the present study, we used network pharmacology to explore the potential active compounds and underlying mechanisms of Dangshen against OS.

After applying the screening methods, we identified 15 active compounds and 48 potential therapeutic targets. The active compounds of Dangshen likely treat OS by regulating these targets. We identified two active compounds, stigmasterol and luteolin, that have been studied previously for their efficacy against OS. Stigmasterol is a phytosterol, which has been shown to exert anticancer, antipyretic, and immune-modulating properties [42–44]. Previously, Trouillas et al. showed that stigmasterol could decrease the proliferation of OS cells [45]. Luteolin is a flavonoid found in vegetables and fruits. It can inhibit the proliferation and induce the apoptosis of OS cells by effectively downregulating the expression of BCL-2, caspase-3, and survivin proteins levels, while upregulating BAX protein levels [46]. In addition, it can induce autophagy in U2OS cells and enhance the sensitivity of these cells to doxorubicin-mediated autophagy signaling [47].

From the therapeutic target-PPI network, the following targets showed larger degree values: TP53, HSP90AA1, CCND1, AR, ERBB2, MDM2, IGF1R, DICER1, CCNE1, and SOD2. These targets may play a major role in the therapeutic effect of Dangshen against OS. Over 70% of OS cases show structural variants or mutations in the TP53 gene [48]. TP53 is a transcription factor that stabilizes following genotoxic stress and induces the transcription of genes associated with cell apoptosis, cycle arrest, and metabolism; thereby, suppressing the development and progression of tumors [49, 50]. HSP90AA1, a 90-kDa heat shock protein [51], is an important target for cancer treatment because it can stabilize several cancer-related client proteins essential for tumor progression, such as AKT, PIM1, and HIF1A [52]. Some studies found that, in tumor biopsies, the absence of HSP90AA1 may serve as a biomarker of favorable outcomes [53, 54]. CCND1 is a member of the cyclin family that encodes cyclin-D1. In addition, it plays a key role in cell cycle regulation [55]. There is substantial evidence showing that CCND1 plays an important role in the development of human cancers [56], including the migration and metastasis of OS [57]. The ERBB family of tyrosine kinases plays an important role in cell cycle regulation, cell proliferation, and cell movement [58]. Tumors that overexpress ERBB2 are less likely to respond to anticancer therapies [59]. Previously, Abdou et al. reported on the overexpression of ERBB2 in OS and its adverse prognostic features, including higher tumor grades [60]. In addition, Wang et al. reported that chimeric anti-caspase-6 and anti-ERBB2 antibodies reduced the metastatic potential of human OS cells [61]. These findings suggest that the therapeutic effect of Dangshen against OS is primarily mediated by cell apoptosis, cell cycle arrest, and the inhibition of tumor cell migration and metastasis.

Next, we performed the GO enrichment analysis and KEGG pathway enrichment analysis of the therapeutic targets. Based on the GO terms, the therapeutic targets showed a strong correlation with the biological processes, such as the G1/S transition of mitotic cell cycle, cell cycle G1/S phase transition, regulation of cell cycle arrest, and the intrinsic apoptotic signaling pathway; cell components, such as cell leading edge, ruffle, ruffle membrane, and endocytic vesicle; and molecular functions, such as p53 binding, disordered domain specific binding, histone deacetylase binding, damaged DNA binding, and ATPase binding. Hence, the mechanism of action for Dangshen may include biological processes, molecular functions, and various cellular components. For example, imbalanced cell cycle regulation is characteristic of tumor cells, and functional defects in cell cycle checkpoints lead to genetic changes that lead to tumor development and progression [62, 63]. In addition, the G1/S phase transition is the target of many anticancer drugs [64]. Apoptosis is a form of cell death that occurs upon receipt of internal or external death signals [65, 66]. In addition, Chaiyawat et al. reported that reduced expression of histone deacetylase-2 of HDAC2 is associated with dismal patient outcomes in OS [67]. Furthermore, Sun et al. reported that histone deacetylase-2 may stimulate the ATM/p53 pathway, leading to DNA damage-mediated cell death in human OS cells [68]. In addition, Cao et al. found that overexpression of histone deacetylase-4 promotes the proliferation and invasion of OS cells [69].

Based on the KEGG terms, the therapeutic targets for Dangshen against OS were primarily associated with the p53 signaling pathway, PI3K-Akt signaling pathway, FoxO signaling pathway, Wnt signaling pathway, and ErbB signaling pathway. P53 plays a critical role in cell cycle checkpoints regulation, DNA damage, and prevention of nonmalignant cells from developing malignant phenotypes [70, 71]. In addition, p53 is an essential regulator of epithelial-mesenchymal transition (EMT) [72], as it promotes the reversal of mesenchymal cells to the epithelial cell phenotype, which reduces the migration and invasion of cells [73]. Many anticancer drugs regulate the p53 signaling pathway. For example, theabrownin triggers DNA damage and induces apoptosis in U2OS cells via p53 signaling activation [74]. Activation of the PI3K-Akt signaling pathway is also associated with cell proliferation and apoptosis of OS cells [75, 76], and the downregulation of AKT reduces cyclin-D1 levels, preventing cells from cycling from G1 to S [77]. The reduced expression of cyclin-D1 also leads to the inhibition of cell proliferation [78]. Simultaneously, AKT downregulates the expression of two essential proteins responsible for apoptosis, caspase-3 and caspase-8 [79]. Abnormal Wnt/*β*-catenin signaling is closely related to the formation, metastasis, and apoptosis of many cancers [80]. The upregulation of Wnt/*β*-catenin signaling was recently observed in OS [81]. As such, the WIF-1 protein, encoded by Wnt inhibitory factor-1 gene, is an important regulatory factor in the Wnt signaling pathway [82]. The WIF-1 gene combines with the Wnt protein to prevent Wnt signaling [83]. Previously, Li et al. reported on the downregulation of WIF-1 in OS cells [84]. Hence, the KEGG analysis revealed that Dangshen produces anticancer effects in OS through the regulation of several proteins, including MDM2, TP53, RAC1, ERBB2, and CCND1, which are all important mediators of various cellular signaling pathways. In addition, most therapeutic targets play their roles in multiple signaling pathways. In addition, most of the therapeutic targets play essential roles in multiple signaling pathways.

Network pharmacology is an analytical method still in development worldwide. However, the method has some inherent flaws. For example, it heavily relies on existing resources of the databases, so it cannot analyze the compounds, targets, or mechanisms that have not been previously explored. Moreover, its predicted active ingredients, targets, and mechanisms of action are all purely theoretical, and there is a lack of experimental verification. Therefore, further clinical investigations are needed.

## 5. Conclusions

In this study, we explored the therapeutic mechanisms of Dangshen against OS through a network pharmacology approach. The therapeutic properties of Dangshen against OS arise from the regulation of biological pathways involved in the proliferation, apoptosis, invasion, and migration of cells, along with DNA damage. We believe these findings demonstrate the importance of understanding traditional Chinese medicines. The current study relied on data mining and analysis, and further clinical investigations are needed to verify the therapeutic mechanisms of Dangshen against OS.

## Figures and Tables

**Table 1:** Characteristics of the active ingredients.

Compound	MF	Structure	MW	OB (%)	DL	HL
Stigmasterol	C_29_H_48_O	fx1	412.77	43.83	0.76	5.57
Stigmast-7-enol	C_29_H_50_O	fx2	414.79	37.42	0.75	6.28
Luteolin	C_15_H_10_O_6_	fx3	286.25	32.00	0.25	15.94
11-Hydroxyrankinidine	C_20_H_24_N_2_O_4_	fx4	356.46	40.00	0.66	10.80
Perlolyrine	C_16_H_12_N_2_O_2_	fx5	264.30	65.95	0.27	12.62
Glycitein	C_16_H_12_O_5_	fx6	284.28	50.48	0.24	16.32
Spinasterol	C_29_H_48_O	fx7	412.77	42.98	0.76	5.32
Frutinone A	C_16_H_8_O_4_	fx8	264.24	65.90	0.34	19.10
Poriferasta-7,22E-dien-3beta-ol	C_29_H_48_O	fx9	412.77	42.98	0.76	5.48
7-Methoxy-2-methyl isoflavone	C_17_H_14_O_3_	fx10	266.31	42.56	0.20	16.89
5-*α*-Stigmastan-3,6-dione	C_29_H_48_O_2_	fx11	428.77	33.12	0.79	5.19
3-*β*-Hydroxymethyllenetanshiquinone	C_18_H_14_O_4_	fx12	294.32	32.16	0.41	22.51
Zinc03978781	C_29_H_48_O	fx13	412.77	43.83	0.76	5.79
Taraxerol	C_30_H_50_O	fx14	426.80	38.40	0.77	2.07
Stigmasterone	C_29_H_46_O	fx15	410.75	45.40	0.76	5.65

**Table 2:** Characteristics of the 48 therapeutic targets.

Target	Name	Degree	Betweenness centrality	Closeness centrality
TP53	Cellular tumor antigen p53	40	0.19161047	0.87037037
HSP90AA1	Heat shock protein HSP 90-alpha	33	0.08748708	0.77049180
CCND1	G1/S-specific cyclin-D1	30	0.05438164	0.73437500
AR	Androgen receptor	29	0.08088557	0.72307692
MDM2	E3 ubiquitin-protein ligase Mdm2	28	0.05726816	0.70149254
ERBB2	Receptor tyrosine-protein kinase erbB-2	28	0.05040772	0.71212121
IGF1R	Insulin-like growth factor-1 receptor	27	0.04394018	0.70149254
DICER1	Endoribonuclease Dicer	24	0.03867798	0.66197183
CCNE1	G1/S-specific cyclin-E1	19	0.01018846	0.61842105
THBS1	Thrombospondin-1	19	0.01547887	0.61038961
SOD2	Superoxide dismutase [Mn], mitochondrial	18	0.01677310	0.61038961
XRCC6	ATP-dependent DNA helicase 2 subunit 1	16	0.02818547	0.59493671
MAPK9	Mitogen-activated protein kinase 9	16	0.01100001	0.59493671
E2F1	Transcription factor E2F1	15	0.00460006	0.58750000
MUC1	Mucin-1	15	0.01251038	0.58024691
CUL1	Cullin-1	14	0.02049293	0.56626506
RAC1	Ras-related C3 botulinum toxin substrate 1	14	0.04760817	0.58750000
B2M	Beta-2-microglobulin	14	0.01143842	0.58024691
MPO	Myeloperoxidase	14	0.01314265	0.57317073
HDAC6	Histone deacetylase 6	12	0.00411776	0.56626506
MAP2K2	Dual specificity mitogen-activated protein kinase kinase 2	12	0.00552768	0.55294118
EZR	Ezrin	12	0.00234926	0.57317073
PAX6	Paired box protein Pax-6	12	0.00636814	0.55952381
TP73	Tumor protein p73	10	0.00045360	0.54022989
BMPR2	Bone morphogenetic protein receptor type 2	10	0.00217280	0.55294118
RPS3	40S ribosomal protein S3	9	0.00443058	0.53409091
HDAC8	Histone deacetylase 8	9	0.00168382	0.54022989
POLB	DNA polymerase *β*	9	0.00620249	0.54651163
FOLH1	Glutamate carboxypeptidase 2	9	0.00471413	0.54651163
SMAD1	Mothers against decapentaplegic homolog 1	8	0.00048566	0.52808989
ATIC	Bifunctional purine biosynthesis protein PURH	8	0.00650462	0.52222222
S100A6	Protein S100-A6	8	0.00018501	0.53409091
NR3C2	Mineralocorticoid receptor	7	0.00397370	0.52808989
EIF2AK2	Interferon-induced, double-stranded RNA-activated protein kinase	7	0.00917844	0.52808989
RPA1	Replication protein A 70 kDa DNA-binding subunit	6	0.00034097	0.51648352
SATB2	DNA-binding protein SATB2	5	0.00026981	0.49473684
CDCA8	Borealin	5	0	0.49473684
FLI1	Friend leukemia integration 1 transcription factor	5	0.00061402	0.50000000
NHP2L1	Human recombinant protein P01	4	0.00020331	0.47000000
CAMK2A	Calcium/calmodulin-dependent protein kinase type II *α* chain	4	0	0.46078431
HSD11B1	Corticosteroid 11-*β*-dehydrogenase isozyme 1	4	0.00067245	0.44761905
ASS1	Argininosuccinate synthase	4	0	0.49473684
FAP	Seprase	4	0.00011563	0.44339623
CANT1	Soluble calcium-activated nucleotidase 1	3	0.00036617	0.44761905
BCL9	B-cell CLL/lymphoma 9 protein	3	0	0.48453608
CSDE1	Cold shock domain-containing protein E1	3	0.00053191	0.41592920
BRD7	Bromodomain-containing protein 7	2	0	0.47959184
SRGAP2	SLIT-ROBO rho GTPase-activating protein 2	1	0	0.37301587

## Data Availability

The data sets generated and analyzed during the present study are available from the corresponding author upon reasonable request.

## Supplementary Materials

Supplementary Materials Description of Supplementary Table S1: the top 50 matched targets for each potential active compound of Dangshen were gathered from PharmMapper, and these targets were regarded as the potential targets of Dangshen. The relationship between the compounds and their matched targets is listed in Supplementary Table S1. Description of Supplementary Table S2: the genes related to osteosarcoma were collected from GeneCards and OMIM databases. The genes related to osteosarcoma are listed in Supplementary Table S2. Description of Supplementary Table S3: the organs with high expression of each therapeutic target were collected via BioGPS. The relationship between the therapeutic targets and their high expressed organs is listed in Supplementary Table S3. Description of Supplementary Table S4: we used the Gene Ontology (GO) database to clarify the possible biological mechanisms. The details of the GO enrichment analysis of biological process are listed in Supplementary Table S4. Description of Supplementary Table S5: we used the Gene Ontology (GO) database to clarify the possible biological mechanisms. The details of the GO enrichment analysis of cell component are listed in Supplementary Table S5. Description of Supplementary Table S6: we used the Gene Ontology (GO) database to clarify the possible biological mechanisms. The details of the GO enrichment analysis of molecular function are listed in Supplementary Table S6. Description of Supplementary Table S7: we used the Kyoto Encyclopedia of Genes and Genomes (KEGG) database to extract biological information about functional classification, annotation, and enriched pathways of therapeutic targets. The details of the KEGG enrichment analysis are listed in Supplementary Table S7. .
